# Plant Ethnoveterinary Practices in Two Pyrenean Territories of Catalonia (Iberian Peninsula) and in Two Areas of the Balearic Islands and Comparison with Ethnobotanical Uses in Human Medicine

**DOI:** 10.1155/2012/896295

**Published:** 2012-07-08

**Authors:** Esperança Carrió, Montse Rigat, Teresa Garnatje, Marina Mayans, Montse Parada, Joan Vallès

**Affiliations:** ^1^Laboratori de Botànica, Facultat de Farmàcia, Universitat de Barcelona, Av. Joan XXIII s/n, 08028 Barcelona, Catalonia, Spain; ^2^Institut Botànic de Barcelona (IBB-CSIC-ICUB), Passeig del Migdia s/n, Parc de Montjuïc, 08028 Barcelona, Catalonia, Spain

## Abstract

This paper presents the results of an ethnobotanical study centred in veterinarian uses in two Catalan Pyrenean regions (Alt Empordà -AE- and High River Ter Valley -AT-, Iberian peninsula) and two Balearic Islands areas (Formentera -FO- and northeastern Mallorca -MA-). In the areas studied, 97 plant species have been claimed to be useful for veterinary purposes. A total of 306 veterinary use reports have been gathered and analysed. The ten most reported plants are *Tanacetum parthenium* (24 use reports), *Parietaria officinalis* (15), *Ranunculus parnassifolius* (14), *Meum athamanticum* (13), *Olea europaea* (13), *Quercus ilex* (12), *Ruta chalepensis* (12), *Sambucus nigra* (10) and *Thymus vulgaris* (10). According to comprehensive reviews, a high number of novelties for plant ethnoveterinary are contributed: 34 species and one subspecies, 11 genera, and three families have not been reported in previous works in this field, and 21 species had only been mentioned once. Several ethnoveterinary uses are coincidental with those in human medicine. Although ethnoveterinary practices are less relevant than in the past in the territories considered, as in all industrialised countries, the knowledge on plant properties and applications is still rich and constitutes a large pool of evidence for phytotherapy, both in domestic animals and humans.

## 1. Introduction

Community animals have always been and continue to be intimately linked to human societies' life. Domesticated (livestock, horses, poultry, other cage food animals such as rabbits, and most pets) and wild animal species (some occasionally captured pets, e.g., certain cage birds) live together with people, who obtain benefits and, at the same time, take care of them. Traditional veterinary practices are documented from as long as 14,000 years ago [1], being at least as ancient as animal domestication [2]. Given the kind of animals dealt with, the rural environment is where such practices (often including healing) are most extended, but pets and other small domestic animals are also quite present in urban areas. This is why ethnoveterinary knowledge is currently in use not only in developing countries, where often no other resources are available, but in developed ones as well, where it constitutes a very valuable complement and/or alternative to the so-called Western veterinary medicine [3]. 

Ethnoveterinary knowledge constitutes a relevant part of ethnobiological knowledge [1]. Data on veterinary plant uses are universally and significantly present in every general ethnobotanical prospection, and even more in those, very frequently, biased to ethnopharmacological aspects. This can be illustrated with a few -out of the very abundant- case examples of monographic ethnobotanical studies in different continents including diverse geographical or thematic approaches and different kinds of societies as regards industrialization level [4–9].

Apart from ethnoveterinary data appearing in general ethnobiological works, a considerable effort has been made to address this subject specifically. All angles of animal health care have been studied, among which those with an ethnographic focus are very relevant. A recent bibliographic compilation [3] provides data from 118 countries all over the world regarding 200 health troubles in 25 livestock species. A great number of papers on ethnoveterinary appear both in ethnobiological and veterinary journals, indicating contemporary interest of the subject in distinct fields. This means that the folk knowledge on animal health problems and the most frequent plant-derived remedies used to treat them are not merely an affair of past times but continue to play an important role in alternative or complementary medicine. To exemplify this we will quote again only an extremely reduced part of the very numerous sources specifically devoted to ethnoveterinary uses and practices, also covering different parts of the world, some of them very general and others focused on a single animal [10–26]. Strictly medical veterinary uses are often complemented with animal feed. In fact, a relatively new return to natural detected in the field of ethnoveterinary (not only referring to traditional ancestral plant use but also to modern uninformed access to plant products as well) has made the border between feed and medicine rather diffuse when addressing health care in animals [15]. In any case, the advanced state of art of ethnoveterinary has already made possible a synthetic work aiming to constitute a worldwide inventory of botanicals for animal health including 451 plant species [27]. All the above-cited general works [4–9] contain, in addition to veterinary ones, data on human medicinal uses, and some of the ethnoveterinary-centred ones also establish the comparison between medicinal uses addressed to humans and animals in the same sociogeographical group [23].

Ethnobotanical studies in Europe -most of them, as already stated, containing ethnoveterinary data- have been and still are abundant ([28] and references therein). Among these, in southern Europe, and in particular in Iberian territories, dealt with in the present paper, specific ethnoveterinary work is not at all scarce, especially in recent times [16, 29–35]. Nevertheless, in Catalonia only two works particularly deal with ethnoveterinary [31, 36], whereas no studies on this subject have been published to date concerning the Balearic Islands. 

The efforts made over the last years to inventory the ethnoveterinarian heritage respond to the fact that industrialisation and rural depopulation have diminished the dependence of people on animals and caused a decrease in traditional animal healing [1] and that the ethnoveterinary knowledge is weak, since it depends exclusively on oral transmission [19]. This weakness is particularly worrying in developed countries, where much more industrial medicine is available and easy to use. Ethnobotanical studies focused on medicinal and on food plants have been previously published from the two Catalan regions considered ([37–39] and references therein), but only very scarce ethnobotanical information is available from the islands of Mallorca and Formentera ([40, 41] and references therein), and ethnoveterinary medicine has not been addressed at all, to date, in any of those territories. Consequent with this situation, the aims of the present work are (1) to inventory plant ethnoveterinary resources in several Catalan and Balearic regions in order to compare the data obtained in insular and continental territories; (2) to evaluate the degree of coincidence of veterinary and human medicinal plant uses in the zones considered; (3) to assess consensus and reliability of these uses and so the vitality of complementary and alternative medicinal practices and their real incidence in the healthcare system.

## 2. Methods

### 2.1. Study Areas

**Figure 1:**
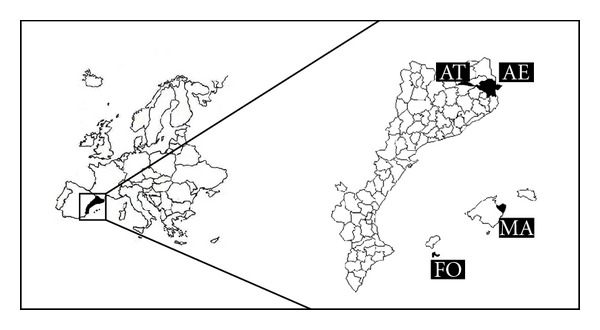
Location of the studied areas in Europe and in the Catalan linguistic area. AE: Alt Empordà; AT: High River Ter Valley; MA: northeastern Mallorca; FO: Formentera.

The territories studied are located in southwestern Europe (Figure 1) and grouped in two close but distinct geographical areas as follows. On the one hand, two regions in the eastern Pyrenees (Catalonia, Iberian peninsula): the district (in Catalan “comarca”) of Alt Empordà (AE), in the foothills of the Pyrenees, and the high mountain area of the High River Ter Valley (AT). On the other hand, two regions in the Balearic Islands: the Artà peninsula area (northeastern) in Mallorca (MA) and Formentera (FO). All these territories share political administration (Spanish) and language (Catalan) and have a common ethnographic and cultural background, with the logical regional nuances.

The Alt Empordà territory comprises 1,358 km^2^ and has around 138,000 inhabitants living in 68 municipalities. The climate is mainly coastal Mediterranean, with a global mean rainfall of 550–750 mm per year and an annual mean temperature of 15.2°C (data from the Catalan Meteorological Service [42]). The most well-known and deep-rooted meteorological phenomenon is a northwesterly wind called the *tramuntana*, responsible for some natural effects, such as some wind-adapted vegetation forms and the desiccation of crops. The district contains an uneven distribution of distinct biogeographical regions—two predominantly Mediterranean ones and also the Eurosiberian in certain mountainous areas [43]. Economically, this area has evolved from an initial agriculture and livestock raising and subsequent industrial forestry exploitation (especially cork) to the more recent tourism and real-estate boom, stronger on the seaside Costa Brava. The High River Ter Valley occupies 294 km^2^ within the Ripollès district. To the North, the valley is limited by peaks that reach almost 2,900 m. The weather is typical of high mountain areas, with cold winters (mean temperature around 3°C) and a mean annual precipitation of 1,284 mm (data corresponding to 2003, Birba, pers. comm.), although the proximity of the Mediterranean Sea softens the climatic conditions. The predominant vegetation belts are the alpine and subalpine [43, 44]. This valley is inhabited by 4,526 people (municipal census, 2004), distributed in 18 population centres belonging to six municipalities. Some of them have suffered an important population decrease, although in recent years houses have been gradually reoccupied as second residences. Agriculture is not very relevant, given its climatic conditions and uneven territory, but many farms and houses within the villages have their own homegardens for private consumption.

Formentera is the smallest of the four inhabited Balearic Islands. It occupies 82 km^2^ and has 9,147 inhabitants (data from 2008) [45] living in nine population centres belonging to one municipality. Its maximum altitude is 195 m, and the climate is Mediterranean with an arid tendency, with an annual mean temperature of 18.6°C and an annual mean rainfall of 370 mm [46]. The main vegetation landscape on this island is the coastal Mediterranean one [43]. Agriculture, timber exploitation, fishing, and salt production have been relevant activities on the island, but nowadays tourism is the most important economic activity. The prospected area in Mallorca is the Artà peninsula, covering the highest part of the Eastern Mountains, in the northeastern corner of the island. It comprises three municipalities: Artà (7,549 inhabitants), Capdepera (11,929), and Son Servera (12,286). One municipality (Artà) belongs to the North district and two municipalities to the Eastern (Capdepera and Son Servera) [45]. The climate in the area is typically Mediterranean (from the coast to the highest peaks, approximately 600 m) with an annual mean temperature of 16.5°C, a mean rainfall of 805.9 mm, and 70% of mean relative humidity [46]. Plant landscapes are basically limited to those of the Mediterranean biogeographic region with a particular relevance of coastal communities [47]. The three municipalities currently share their main reliance on tourism, having left aside the primary sector of agriculture and livestock, which used to be the main activities throughout their history. Currently the percentage of active people engaged in this economic sector (data from 2010) [45, 48] is only 1.24 for the whole island, and a relevant part of peasants are retired people who continue to cultivate some extension of land not far from the place where they live.

### 2.2. Informants and Periods of Field Work

The information was obtained from people either born and (almost) permanently located in each concerned territory or having lived there most of their life. The informants' selection has been basically done on a snowball basis, mostly starting with people known by the authors or by some authors' friends or relatives. All the authors of the present paper have been born in and live in or have links and frequent contacts with one of the studied territories, which facilitated the approach to the informants. A special emphasis has been made in contacting older people, since we supposed them to possess a higher amount of traditional knowledge due to the years of experience and the possibility of remembering pretouristic times, although young people have also been taken into account. In AE 101 interviews with a total of 179 informants (71% women, 29% men, maximum, minimum, and mean ages 95, 23, and 71 years, resp.) were carried out from 1995 to 2008. In AT 42 interviews concerning 60 informants (63% women, 37% men, maximum, minimum, and mean ages 87, 45, and 71 years, resp.) were performed in 2004 and 2005. In FO, 12 interviews were performed with 14 informants (78.6% women, 21.4% men, maximum, minimum, and mean ages 90, 68, and 80 years, resp.) in 2010 and 2011. In MA 42 informants (40% women, 60% men, maximum, minimum, and mean ages 99, 28, and 77 years, resp.) were interviewed between 2005 and 2011.

### 2.3. Ethnobotanical Interviews

The conversations were recorded, and pictures were taken during their development, all this with the permission of the informants. We did not use a closed questionnaire and avoided as much as possible asking direct questions, so as not to coerce the interviewees and so diminish their spontaneity. We used a combination of what the ethnographers call nonstructured or nondirected interview and the model termed as structured, direct, or focused interview [49], the latter called semistructured in most ethnobiological literature [50]. In some cases we also practised what the above-cited authors termed group interview, but those with only one informant constituted the majority. Since, as already stated, the authors live in or go frequently to the study areas, participant observation [50, 51] has also been conducted in a large number of cases. Most times more than one conversation with an informant was performed. During the interviews we asked the informants, in addition to their providing data on plant knowledge, to tell us how, when, and where they collected the plants, how they kept them, and how they prepared them for use. One of the principal points in our interviews being medicinal plants, an effort has been made to steer the conversation not only towards comments relevant to human medicine but also touching the health and treatment of domestic animals. So, we asked the informants about plants traditionally used in the area for treating animal illnesses.

### 2.4. Plant Collection and Identification

Plant materials of all taxa mentioned were collected according to the advice and recommendations of the informants and, whenever possible, together with them. They were identified using the *Flora dels Països Catalans* [52], the *Flora Manual dels Països Catalans* [53], and counting, in some cases, on the help of specialists in floristic and systematic botany. For foreign or cultivated species determination we followed [54, 55]. Vouchers are deposited in the herbarium BCN (*Centre de Documentació de Biodiversitat Vegetal, Universitat de Barcelona*).

### 2.5. Data Analysis

Data collected were introduced and analysed using a database we had designed [56] to ensure an organized pool of the gathered information from interviews. This permitted the standardisation of data entry and further analysis. This database has been designed as an open source interface, a constantly growing platform for ethnobotanical data collected within Catalan-speaking territories. Technical characteristics of the database are a MySQL server, read on php format and data exported as csv. With comparison intentions, we made an analysis of the coincidences and the degree of novelty between our own data and data from bibliography on ethnoveterinary plant uses in different areas (see the literature quoted in the introduction, especially [27], which constitutes a checklist of world ethnoveterinary plants).

Several quantitative ethnobotany indices accounting for the relevance and/or reliability of folk plant knowledge have been calculated for the ethnoveterinary plant uses in the territories studied: ethnoveterinaricity index (EvI), an adaptation of Portère's ethnobotanicity index (EI, [57]) to veterinary plant uses; informant consensus factor (F_IC_, [58]); cultural importance index for each species and for all the territories (CI, [59]), on the basis of all informants, having or not provided veterinarian information. Also, the Jaccard's similarity index [60] has been calculated from the matrix of all use reports (for the four areas) using R software [61], and its visualisation has been designed as a PCA (principal component analysis) plot. This plot is complementary to a 4-term Venn diagram [62] that compares the number of plant species shared (one-to-one and by groups) among studied territories. Statistical analyses were carried out using XLSTAT 2009 v.3.02 (Addinsoft Corporation) available for Microsoft Excel 2003. Descriptive statistics (including rank, mean, and standard deviation, among other parameters) have been calculated for all the studied variables. One-way ANOVA has been conducted in order to test the differences, if any, in the CI among three studied areas (MA and FO are grouped to avoid a sampling bias). Least significant difference (LSD) test was carried out after ANOVA analysis to identify which pairs are significantly different. Chi-square (*χ* ^2^) tests are used to compare the parameters (part of plant, pharmaceutical form, etc.) among studied areas.

## 3. Results and Discussion

In the Catalan and Balearic areas studied, 97 plant species (101 taxa to the levels of subspecies and variety; 49 in AE, 49 in AT, 11 in FO, 17 in MA) have been claimed to be useful for veterinary purposes.  Table 1 presents the plants recorded, grouped in alphabetical order of genera, with indication of scientific and local Catalan names, herbarium voucher number, botanical family, part used, pharmaceutical form, administration way, and veterinary and human uses.  Table 2 summarizes numerical information on the informants and the territories studied, the plants used, reports, local names, families and related data, and some quantitative ethnobotany indexes calculated for these plant uses in the areas prospected.

Apart from the plants used against animal diseases, a considerable percentage of plant species are employed in the different areas as fodder: 21% (AT), 73% (AE), 16% (MA), and 14% (FO). This paper being specifically devoted to medicinal uses, we did not consider all feed plants as having an ethnoveterinary application. Anyway they, too, contribute to animal health, and in many cases the informants attribute them with medicinal properties complementary to the nutritional effect. These plants fit within the category of folk functional foods, proposed by Rigat et al. [37] to include plants traditionally used as nutraceuticals or food medicines, terms usually applied in human medicine, but perfectly transposable to veterinary medicine. As Pearson [15] remarked, there is a frequent possible confusion between feed and drug in ethnoveterinary.

The number of veterinary plant taxa (101) is intermediate between those recorded in the two precedent investigations on this subject in the Catalan cultural area (89 in Montseny [31], 195 in Pallars, Pyrenees [36]). It also occupies a medium position in a ranking going from 36 to 280 taxa used for animal health care in European, African, Asian, and American territories [12, 18, 19, 22, 23, 33–35]. In fact, it is not far from the average of the data contained in the 10 studies reported in the preceding lines (121.3 average taxa for the 11 studies), representing a large geographical range and up to a third of the 451 taxa collected in a world checklist of veterinary botanicals [27]. The big differences among plant number in these areas may be attributed, apart from geographical and possible cultural facts, to the different extension of the territories prospected (from small communities to entire countries). In any case, we can consider the number of plant taxa reported in the present study as rather high, taking into account the decrease in folk animal health practices experienced in industrialised areas [1].

 The ten most reported plants were *Tanacetum parthenium* (24 use reports), *Parietaria officinalis* (15), *Ranunculus parnassifolius* (14), *Meum athamanticum* (13), *Olea europaea* (13), *Quercus ilex* (12), *Ruta chalepensis* (12), *Sambucus nigra* (10), *Thymus vulgaris* (10), and *Malva sylvestris* (9). Among these plants, there are some of the most reported also in other Mediterranean territories, especially *Malva sylvestris*, *Parietaria officinalis*, *Ruta chalepensis*, *Sambucus nigra*, and *Thymus vulgaris* [18, 30, 31, 33, 35, 36]. An originality of this study is the report in top position in the ranking of *Meum athamanticum* and *Ranunculus parnassifolius*. These two central European high mountain plants [53], reported, respectively, for the second and first time in veterinary (see Section 3.2 and Table 1), are much appreciated in one of the regions studied (AT), the first one as postlabour coadjuvant and the second one for different kinds of uses, antineoplastic included. Another high mountain *Ranunculus* species (*R. aconitifolius*) has been recorded from Occitan shepherds of a French Central Massif region with a use (resolutive) coincidental with one of those of *R. parnassifolius* in the studied area [26]. It is worth mentioning also the plant occupying the 11th position in our list as per number of reports, *Eryngium campestre*. This plant, only reported in veterinary to date with the same use in another Catalan region [31], and with different uses in Andalusia [34] and Aragon [63], is widely employed in two of the areas prospected (AE, AT) as an antiophidian. In addition, another species of the same genus (*E. bourgatii*, not reported to date in veterinary) occupies the 13th position in the list also with the same use. These two taxa were not recorded (as another congeneric one, *E. foetidum*, was) in a comprehensive checklist of plants used against snake bite, containing 773 taxa [64], and only one reference to an undetermined *Eryngium* sp. is provided in a work on Turkish ethnoveterinary [65].

The families containing more taxa with claimed veterinary uses are Lamiaceae (10 taxa), Asteraceae (9), Apiaceae (6), Liliaceae (6), Pinaceae (6), and Crassulaceae (5). Some of them (Apiaceae, Asteraceae, Lamiaceae, Liliaceae) are at the same time large families and typically abundant in Mediterranean areas, and they are among the more represented families in most ethnobotanical works in this biogeographical region ([66] and references therein). Another one, Pinaceae, not so big in terms of number of taxa, is landscape dominating in significant parts of the studied areas. All these families but one (Crassulaceae) are among the top ten in the recent world inventory of veterinary ethnobotany [27]. Most of these families are coincidental with the main ones appearing in other studies in the Catalan linguistic area [31, 36], as well as in other Iberian [33–35] and other Mediterranean [18, 30] territories. In an area within Argentina, a great distance from those here studied, the cosmopolitan families Asteraceae and Lamiaceae are coincidental as some of the most reported ones, but others, such as Verbenaceae and Zygophyllaceae, make a difference [23]. Similarly, in a South African region Asteraceae also occupy a preeminent place, but not Lamiaceae, whereas Capparaceae and Euphorbiaceae are particularly relevant [19], contrarily to the currently considered area. More differential families may be recognized in a study performed in an Indian territory, where even the Asteraceae do not appear and the Lamiaceae are only represented by one report among results given for 17 families [17]. This predominance as more reported families of those large and with many representatives in the flora of the area considered agrees with the statement of Johns et al. [67] that the closer to the user a plant grows, the more it is employed. We could also verify this point in many works on folk plant uses in human medicine ([68] and references therein). Of course, the presence of well-known medicinal plants in all these particularly relevant families goes in the same sense. The Crassulaceae, located in position 23 in this catalogue and with only 39 taxa present in Catalan language territories (an area much larger than the one we cover) [54], constitute a particularity of the studied zone. One cultivated/subspontaneous and four wild taxa of four genera belonging to this family are profusely used (11 reports), mostly externally, for wounds and dermatologic affections, although the internal use of *Aeonium arboreum* on the island of Formentera (three reports) as a hens' egg calcifier (i.e., to add calcium and so to reinforce the eggshell) should be highlighted.

### 3.1. Comparison among the Studied Territories

**Figure 2:**
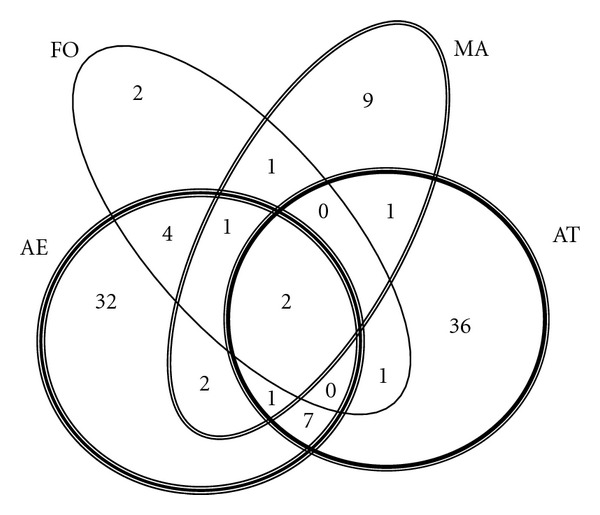
Venn's diagram showing the coincidences in plant species used in the four territories studied. See Figure 1 caption for abbreviations.

In this section, graphical information and descriptive statistics are presented in order to give a detailed interpretation of the comparison among prospected territories. On the one hand, and according to the 4-area Venn diagram of Figure 2, only two species (in the very centre of the graph) have been cited in all the territories; these are *Olea europaea* var. *europaea* and *Ruta chalepensis*. If we look, on the one hand, at the Venn diagram, we notice that the information from Alt Empordà and High River Ter Valley is nearly independent in itself, but Formentera shares almost all the uses and plant species reported (9 shared versus its total of 11). Meanwhile, Mallorca appears in the crossroad in between the three other areas, and nearly half of all the species cited by Mallorcans (9) are different from the species of the other places studied. Notwithstanding, there are 8 plant species cited in Mallorca uniformly distributed among the rest of the areas. No plant species coincidences were found among the groups AE-AT-FO and AT-MA-FO, probably because of the lesser data from Formentera included in the analysis.

**Figure 3:**
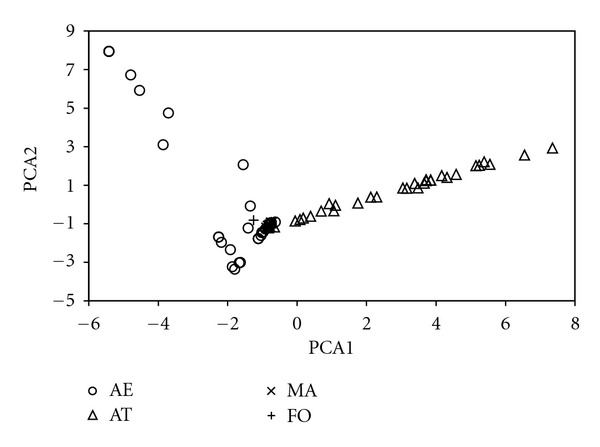
Principal component plot showing the similarities between use reports in the four studied territories. See Figure 1 caption for abbreviations.

 On the other hand, if we look at the PCA plot (referring similarities between use reports (Figure 3)), we also find a convergence zone where several use reports from the four areas coincide. The condensation of Mallorcan data at this point could be explained due to the eight largely distributed species above mentioned, as well as the data from Formentera, which appear close to this area for sharing many of its taxa with the other regions. Moreover, the AE data spread distribution of the PCA plot, as opposed to AT, may be explained because many use reports are cited by a unique informant, so that the similarity line is upward deflected. In short, PCA plot reveals that, considering not only plant species but UR, islands have ethnobotanical similarities and there are shared UR citations with the four regions considered in the study.

 One-way ANOVA and LSD test show statistically significant differences in the CI among four studied areas (*P* < 0.05) excepting for AT versus MA (*P* > 0.05). When MA and FO are grouped, no significant differences between these two Balearic Islands and AT were found (*P* > 0.05). Chi-square tests also show differences between observed and expected frequencies in all studied areas for the three variables included: part used (*χ* ^2^ = 199.34  df = 44  *P* < 0.0001), pharmaceutical form (*χ* ^2^ = 211.96  df = 48*P* < 0.0001), and veterinary uses (*χ* ^2^ = 338.77  df = 118  *P* < 0.0001). 

The comparison of ethnoveterinary data from the four areas leads us to consider that the common heritage of plant uses (and specifically for veterinary treatments in the present work) throughout the Catalan-speaking territories is nuanced by local features. It has to be emphasized that this is the first cross-regional ethnobotanical comparison made up with Catalan data. Similarly to other comparative studies using coordinated methodology and dealing with ethnobotanical data [18, 68, 69], it is very difficult to assure that there is a standard traditional veterinary knowledge among the four areas without contemplating floristic, bioclimatic and sociohistorical aspects.

### 3.2. Comparison with Previous Reports and Novelty in Uses in the Regions Studied

A certain number of plant species and plant uses are new or very scarcely previously reported in ethnoveterinary. We have first compared our results with a recent world catalogue of plants used in this field [27], built with information from 222 publications and including data on 451 taxa at specific or subspecific levels, 308 genera and 116 families. From this comparison, we found that 42 species and one subspecies, 17 genera and five families do not appear in this inventory and must thus be considered new or very scarcely reported as useful in veterinary. In addition, 27 taxa are also not very commonly used in veterinary, since they were reported only once in the world inventory, 17 of them with data coming from a previous work of our team in Montseny [31], an area belonging to the same cultural community of the currently considered zones. These new or rarely recorded taxa have been crossed with a review of plants used in ethnoveterinary in Italy ([30], not quoted in [27]), containing information on 280 species or infraspecific taxa belonging to 71 families. In this review, eight species, six genera, and two families not listed in [27] appeared, as well as six of the species cited only once. Thus, the novelties contributed in the present paper are 34 species and one subspecies, 11 genera, and three families, plus 21 species only mentioned once. Irrespective of the wide reach of the two reviews considered, it is sure that the amount of new taxa could be reduced with a still more comprehensive literature cross (e.g., four plants reported in this study and only previously cited once in veterinary according to the above-mentioned sources have been very recently mentioned in Andalusia [34], and one, *Meum athamanticum*, not appearing in any of the checklists mentioned was already reported by Font Quer in 1961 [70]). Nonetheless, we believe that in any case the number of taxa of different taxonomic level not, or very rarely, previously reported as used in veterinary is significant. The comparatively small amount of work on ethnoveterinary in Europe could contribute to explain this high level of new information.

### 3.3. Parts of Plant Used, Preparation and Administration Forms, and Plant Use Categories

**Figure 4:**
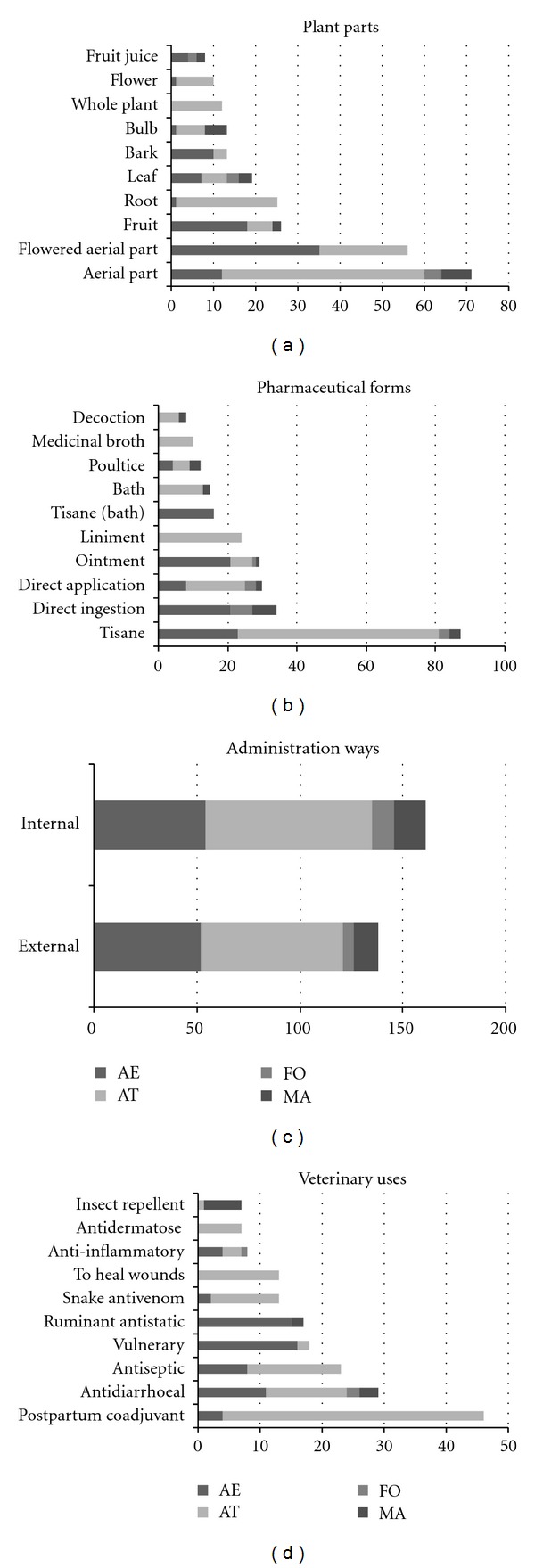
Summary of the top ten used plant parts, preparation and administration forms, and of the top ten veterinary uses in the four territories studied. See Figure 1 caption for abbreviations. The figures in the graphics mean number of use reports.

A summary of the top ten used plant parts, preparation and administration forms, is graphically represented in Figure 4. This figure also includes the ten most cited veterinary use categories, which are compared to human medical indications in Section 3.5. The plant parts most commonly used for veterinary remedies preparation, concerning the general overview of the four areas, are aerial parts (leaves and stems; 71 reports), flowered aerial parts (44 reports), fruits (26 reports), roots (25 reports), and leaves (19 reports). However, it is outstanding that only aerial parts and leaves are represented in the four areas, meaning that leaves (alone, or together with the stems in which they are inserted) are the most popular organ in terms of geographical extension. These numbers do not differ much from other ethnoveterinary studies [31, 33–35] neither do they from human ethnopharmacological works for the same areas [66, 71, 72], where aerial parts and leaves are at the top of list of plant part analysis. The percentage of internal administration form (54.03%) is not much higher than the external (45.97%), but still tisanes are the preferred preparation method (with 86 reports) for animal traditional therapies. Tisanes are not difficult to prepare but, after tisane, we count the direct ingestion (34 reports) and direct application (31 reports), which are even easier ways to treat animals (most of them are grass-eating domestic animals). With particular regard to excipients—apart from water—olive oil has to be counted as the most important in the four areas. The use of olive oil ointments for external administration appears in a fourth place in the preparation classification, and it is especially formulated as vulnerary, cicatrizing, and against dermatologic ailments.

The most cited veterinary use category as an absolute value for all the territories altogether is the postpartum coadjuvant. However, this is not a significant set since there are 42 reports out of 46 that have been collected for the AT area. For the whole area prospection, the most representative veterinary indication is the antidiarrhoeal. Indeed, plants aimed to treat gastrointestinal disorders are frequently on the top of the latest ethnoveterinary usage lists [23, 34, 35]. Top veterinary uses concerning every study territory separately are diverse enough: vulnerary for AE, postpartum coadjuvant for AT, egg calcifier for FO, and insect repellent for MA. The reason of these differences may lie on the type of livestock treated; for example, results from Mallorca have a socioeconomic bias on sheep treatment since these animals have historically been the first islander meat resource, well ahead of the pig [73]. The treatment of sheep against fly larvae explains that many plant citations have been made for insect repellent.

### 3.4. Quantitative Ethnobotany: Indices Assessing the Importance, Persistence, and Reliability of Veterinary Plant Uses in the Regions Studied

The ethnoveterinaricity index (EvI), which we have defined here adapting the classical ethnobotanicity index to include only use reports of plants concerning animal health, is low in all the studied areas. It oscillates between 0.02 and 0.03, this meaning that only around 2-3% of plants present in these territories are claimed to be useful in veterinary. General ethnobotanicity indices or indices referring to all medicinal plant uses in Mediterranean territories oscillate between 0.05 and 0.51, the average value for 21 territories being 0.22 [66]. The percentage of plants used in veterinary is, logically and in all cases, lower than general ethnobotanicity indices. In the present case, it is also lower than in the two previous reports on Catalan ethnoveterinary (0.06 in Montseny [31] and 0.13 in Pallars [36]), but similar to that recorded in another Iberian area (0.02 in Navarra [33]).

The mean informant consensus factor (F_IC_) considering the four areas studied is 0.67, and it ranges from 0.33 to 0.68, being bigger in the two Catalonian areas (AE 0.54, AT 0.68) than in the Balearic ones (FO 0.33, MA 0.40). This index, with the maximum value of 1, shows the consistency of uses among the informants of a given territory, and thus it is one of the indicators of reliability for such uses. The values of the Catalonian areas are close to that from Montseny, 0.66 [31]. In general, F_IC_ values for ethnoveterinary are clearly lower than those of works on human medicinal uses in the same territories (AE 0.91, AT 0.87, MA 0.71, FO 0.73) ([66, 71, 74], Mayans, Carrió, and Vallès, unpubl. res.). This suggests a preeminence of human medicine over veterinary, at least in current times, in the society prospected: veterinary uses are less homogeneous and consistent that human medical ones, since they are in fact perceived nowadays as less relevant, less necessary. In any case, it is interesting to remark that the ethnoveterinary F_IC_s are proportional to the general (for all medicinal plant uses) ones in each territory, confirming the above-described fact. Most works on ethnobotany of veterinarian plants do not mention F_IC_ values. The ones reported for eight zones of Navarra (Western Pyrenees, Spain) range from 0 (in the main cities areas) to 0.63, the mean being of 0.37 [33]. These values are slightly lower, but similar to those recorded in the present paper; in Navarra, the F_IC_ for general medical ethnobotany is also higher than the veterinary one (0.65 [75]). Finally, it is worth mentioning that F_IC_ of the whole territory studied (0.67) are lower but not very far from those for human medicinal uses in some Mexican and Indian regions (0.75, 0.79 [76–78]). The latter were recorded in countries where folk medicinal plant knowledge is considered relevant and currently in use. To summarize, there is not a very pronounced agreement in ethnoveterinary plant uses, but neither it is extremely low, when taking into account the indicative figures from other territories and the current relevance of domestic animals along with the modern way to address their health troubles. 

Another way to assess the relevance of folk plant uses is the recently described cultural importance index (CI, [59]). The global values of this index are low in the areas prospected (Table 1), ranging from 0.01 to 0.02. This is not surprising taking into account that in each territory, and in all of them together, the number of plants claimed as useful is high, and for many of them a scarce number of reports has been collected. In addition, even the interviewees who did not report any veterinary use are counted. The CI ranges per area, also calculated on the basis of all informants, not only those having provided veterinarian information, are slightly or clearly—depending on the cases—higher: 0.06–0.08 (AE), 0.02–0.27 (AT), 0.07–0.21 (FO), and 0.02–0.07 (MA). Just to give an example of the different CI values in a veterinary and in a human medicinal ethnobotanical survey, *Santolina chamaecyparissus* in MA has 0.07 for ethnoveterinary and 0.81 for human pharmaceutical ethnobotany [74], this indicating the much higher number of informants that report medical uses as opposed to those claiming veterinary ones. This index was rather designed to highlight the most relevant plants used for a specific purpose in a particular cultural area; this is why the most reported plants bear the larger indices. This index assesses the relevance of a plant in a culture. Here culture can be understood either in its broad sense (in our case, the Catalan one, common to all studied areas) or in its restricted sense, distinguishing in the present case high mountain (AT), medium mountain and plain (AE) and insular (FO, MA) cultures. All these cultures in a more restricted sense are shaped on the one hand by belonging to a linguistic community and on the other hand by geographical and socioeconomic conditions: as stated above (Section 3.1); the common base is modelled by and diversified with local specificities. The top ten plants regarding report number, quoted in the first paragraphs Section 3, have, logically, the highest CIs in the whole area studied, but some plants not present in this list bear the highest CIs in particular territories, such as *Aeonium arboreum* (FO), *Allium sativum*, and *Santolina chamaecyparissus* (MA). The description of this index is recent, and so very few papers include it in their analyses. Nevertheless, an ethnoveterinary study of an Iberian area, Arribes del Duero (Salamanca, Western Spain), also shows a high number of low CIs (with a minimum value of 0.04), but at the same time some very high, four of them higher than 1.

### 3.5. Comparison between Veterinarian and Human Medicinal Plant Uses

Table 1 shows the agreements between veterinary and human medicinal plant uses in the areas studied, meaning the strict coincidence not only of the plant employed but of its claimed properties as well. The number of total human and veterinary medicine uses is presented in Table 2. There are of course many more human medicinal uses different from those addressed to animal health ([66, 71, 74], Mayans, Carrió, and Vallès, unpubl. res.), but we have highlighted here only the uses that are common to both people and animals.

Table 2 shows that the number of plants reported to be useful in veterinary medicine is dramatically low as compared with that of those indicated to be used in human medicine in the same territories (15.3% in AE, 21.8% in AT, 12% in FO, 14.5% in MA). Yet the proportion of informants who reported veterinary uses is low, also in every area prospected (absolute figures in Table 2; 25.7% in AE, 68.3% in AT, 28.6% in FO, 31% in MA). Facing this situation, the question arises as to whether it could be the consequence of a bias in data recording. It is true that when talking about medicinal plants (one of the main focuses in our ethnobotanical interviews), it is implicitly clear for both interviewers and interviewees that human medicinal uses have to be addressed, whereas the reference to veterinary uses is not so evident. So, we must admit a slight weight of this factor in this difference between human and animal medicinal plant use reports. Nevertheless, in many cases in which the point of ethnoveterinary uses is explicitly present in the conversations, this does not significantly increase the information on animal health care. In addition, the number of veterinary plants recorded in the areas prospected is not lower than those published for other studies in the same biogeographical region [18, 31, 33–36]. Moreover, 68.3% of AT informants provided ethnoveterinary data as compared with 25.7% in AE, but the increase of information only represented 6.5%. We believe that the decline in human dependence on domestic animals in so-called western societies explains basically the unbalance between both kinds of medicinal plant uses. In this sense, it is interesting to remark that AT (the territory studied with a larger proportion of informants supplying ethnoveterinary information and of veterinary uses recorded) is a high mountain area in which domestic animals still play a significant role, at least more than in the other places.

In any case, the 306 veterinary use reports of 101 taxa in the four areas considered constitute a large therapeutic corpus, with a not insignificant part in agreement with human medicinal uses also claimed by the informants. The proportion of coincidental human and animal plant-use categories in all the studied areas is 42.9%, ranging from 30.1% to 52.2% in the different territories (Table 2). In terms of number of reports of the same use of a plant in human and animal health, the figures are also high (Table 2), representing a 52.7% of agreement when all areas are considered together, and being of 31.1% in AE, 69.8% in AT, 25% in FO, and 59.3% in MA. Again, AT is the territory with the highest coincidence in use categories and reports, this indicating a still important degree of validity of veterinary practices in this area since the more animal health care uses persist, the more they may coincide with those for human health troubles, in general more operative and easily recalled.

In most cases, the plants for which human medicine reports are coincidental with veterinary ones are among the most commonly used to address people's ailments. As an example, in MA, four of the plants so considered (*Allium sativum*, *Citrus limon*, *Herniaria hirsuta*, and *Santolina chamaecyparissus*) appear in the list of the top five medicinal species in the area. In these cases plant preparations for animals are often similar to those for humans [74], showing on the one hand how important animals were in times gone by and on the other hand the proximity of veterinary and human medicine and so the relevance of ethnoveterinary data as evidences for phytotherapy in general. In AE the panorama is similar (present data and [66]) to one of the top species (*Allium sativum*) coincidental with MA.

Two use categories in which there is a convergence of veterinary and human medical uses are gastrointestinal and skin troubles. On the one hand, digestive, antidiarrhoeal, gastrointestinal antialgic and anti-inflammatory are uses corresponding to usually nonsevere chronic illnesses very often treated with folk phytotherapeutic remedies ([34, 66] and references therein). On the other hand, skin affections may also be nonsevere troubles (such as warts) and have a particular incidence in rural societies, in people dealing with agricultural and livestock-raising activities (wounds and some kinds of skin infections), this kind of affection being almost as common in humans as in the animals they take care of. Conversely, a use category that was once shared by people and domestic animals, labour and postlabour coadjuvant, is now almost exclusively restricted to animals, basically cows. The explanation is evident: the medical assistance in labour has increased dramatically more in human beings than in livestock, apart from the fact that many labour coadjuvants may have abortive effects if used in a nonadequate manner or period of time, and there is a higher vigilance of this aspect in people than in domestic animals.

### 3.6. Concluding Remarks

Our research in four European areas has verified that, as Mathias et al. [3] wrote, ethnoveterinary practices constitute viable alternatives or complements to conventional, Western-style veterinary medicine. The collection of information on ethnobotanical uses of plants in veterinary medicine, as done in the present work, is the first step of the process that can permit the passage from folk, often small-scale, uses to industrial or at least medium-scale applications. It is undoubtedly one of the beneficial and appropriate ethnoveterinary interventions that, in words of Wanzala et al. [1], represent a major challenge in the development of this discipline in the 21st century. Muhammad et al. [16] stated that these data provide a basis for further validation of practices and plant uses in the context of a professional approach to ethnoveterinary medicine. Additionally, we stress that recording these data is already in itself a part of this validation, since it provides scientific evidence of plant uses, after which, chemical, pharmacological, and other issues should be addressed. Some examples of legislation and herbal products development in western Europe [82] make us believe that further ethnobotanical studies in the field of veterinary are needed, followed by a coordination with different stakeholders (livestock raisers, veterinary surgeons, chemists, health policy managers and deciders, pharmaceutical firms, among others) in order to integrate ethnoveterinary knowledge—as we have seen, closely related to human ethnomedicinal one—in health policies.

 As for all domains of ethnobiology, the inventory of ethnoveterinary practices is urgent, mostly in industrialised countries. Concerning specifically animal health care, in relatively few years we have passed, at least in southwestern Europe, from a lifestyle in which, according to a popular saying, the illness of the mule was considered worse for a rural family than a trouble in a member of the family to a situation of almost no dependence on domestic animals and from the practical absence of veterinary doctors and industrial medicines to the inverse situation even in the smaller population nuclei. The popular saying regarding the mule, obviously an exaggeration, can still be heard amongst elderly people in the regions prospected, but today the situation is different. Thus, a certain amount of ethnoveterinary knowledge in the areas described is no longer in practice and must be collected—not only as a cultural and biological heritage, but also as possible sources for new drugs for animals and humans—before it is too late.

## Figures and Tables

**Table 1:** Plants with veterinary medicinal uses claimed in the areas studied. VR: veterinary reports; HR: human medicine reports (only those coincidental with veterinary ones); CI, CIg: cultural importance index [54] for each area and in all the territories studied (global); AE: Alt Empordà; AT: High River Ter Valley; MA: northeastern Mallorca (Mallorca); FO: Formentera. Asterisks (*) indicate the subspecies, species, genera, and families not reported as useful in veterinary in [27, 30], and crosses (^+^) indicate the subspecies, species, genera, and families reported only once as useful in veterinary in these quoted works (both signs are placed after the name of the category concerned).

Scientific names (family, voucher specimen)	Catalan names	Part used	Pharmaceutical form	Administration way	Veterinary use	VR	HR	Area (CI, CIg)
*Achillea ptarmica *L* subsp. *pyrenaica *(Sibth. ex Godr. in Gren. et Godr.) Heimerl (Asteraceae, BCN 24701)	camamilla, camamilla de muntanya, camamilla de Rojà	Inflorescence	Tisane (with *Sambucus nigra*)	Internal	Antidiarrhoeal	1	0	AT (0.05, 0.01)
			Tisane (with sodium bicarbonate and olive oil)	Internal	Laxative	1	0	AT
			Tisane (with *Thymus vulgaris* and *Tilia platyphyllos*)	Internal	Salutiferous	1	1	AT

Aeonium* arboreum (L.) Webb and Berthel. (Crassulaceae, BCN 81566)	consolva	Stem	Direct ingestion	Internal	Egg calcifier	3	0	FO (0.21, 0.01)

*Agave americana* L. (Agavaceae^+^, BCN 46860)	figuerassa grossa, figuerassa de marge	Leaf	Direct application	External	Vulnerary	1	0	AE (0.006, 0.003)

*Agrimonia eupatoria* L.^+^ (Rosaceae, BCN 29619)	gremònica, tapaculs	Leaf	Tisane	Internal	Antidiarrhoeal	1	1	AE (0.006, 0.003)

*Allium cepa* L. (Liliaceae, BCN 28655)	ceba	Bulb	Tisane	Internal	Laxative	1	2	AE (0.006, 0.003)
			Direct ingestion	Internal	Anticatarrhal (fowl pip)	1	2	MA (0.024, 0.007)

*Allium sativum* L. (Liliaceae, BCN 24708)	all	Bulb	Direct application	External	For stings	2	2	MA (0.071, 0.02)
			Direct ingestion	Internal	Salutiferous	1	1	MA
			Liniment (with *Senecio vulgaris*)	External	Anti-inflammatory (antigotose)	1	1	AT (0.017, 0.02)
			Liniment (with *Bryonia cretica* or *Umbilicus rupestris*)	External	To heal wounds	2	2	AT

*Aloe vera* (L.) Burm.f.* (Liliaceae, BCN 27242)	aloe vera	Leaf	Direct application	External	Bone reinforcer	1	0	MA (0.024, 0.003)

*Althaea officinalis* L. (Malvaceae, BCN 24709)	malví	Root	Liniment (with *Bryonia cretica* or *Umbilicus rupestris*)	External	To heal wounds	1	1	AT (0.017, 0.003

*Alyssum maritimum* (L.) Lam.* (Brassicaceae, BCN 29622)	herba fetgera	Flowered aerial part	Direct ingestion	Internal	Antiicteric	1	0	AE (0.006, 0.003)

*Arum italicum* Mill.^+^ (Araceae, BCN 32358)	xèrria	Fruit	Ointment	External	Anti-inflammatory	1	1	AE (0.006, 0.003)

*Arundo donax* L.^+^ (Poaceae, BCN 24720)	canya	Root	Tisane	Internal	Unknown	1	0	AT (0.017, 0.003)

*Avena sativa* L. (Poaceae, BCN 29839)	civada	Aerial part	Direct ingestion	Internal	Blood pressure regulator	1	0	AE (0.006, 0.003)

*Beta vulgaris* L.^+^ subsp. *vulgaris* (Chenopodiaceae, BCN 24724)	bleda	Leaf	Medicinal broth	Internal	Postpartum coadjuvant (emollient)	2	0	AT (0.033, 0.007)

*Brassica oleracea* L.^+^ subsp. *oleracea* (Brassicaceae, BCN 32181)	bròquil	Flowered aerial part	Tisane	Internal	Antidiarrhoeal	1	0	AE (0.006, 0.003)

*Bryonia cretica* L.^+^ subsp. *dioica* (Jacq.) Tutin (Cucurbitaceae, BCN 24730)	carbassí, carbassina	Root	Bath	External	Antiparasitic	1	0	AT (0.07, 0.01)
			Liniment (with *Umbilicus rupestris*)	External	To heal wounds	2	1	AT
			Poultice	External	Antidermatose	1	0	AT

*Calendula officinalis* L. (Asteraceae, BCN 24732)	calèndula	Flower head	Essence	External	Antitoxic	1	1	AT (0.017, 0.003)

*Capsella * ^+^ * bursa-pastoris* (L.) Medic. (Brassicaceae, BCN 24736)	bossa de pastor, caps blancs	Flowered aerial part	Tisane	Internal	Postpartum coadjuvant (antihemorrhagic and antihypertensive)	1	1	AT (0.017, 0.003)

*Capsicum annuum* L. (Solanaceae, BCN 24737)	pebre coent	Fruit	Maceration in oil	External	Insect repellent	2	0	MA (0.05, 0.007)

Centaurium* erythraea Rafn subsp. *erythraea* (Gentianaceae^+^, BCN 29849)	herba de santa Aura	Flowered aerial part	Tisane	Internal	Hypertensive	1	0	AE (0.006, 0.003)

*Ceratonia siliqua* L.^+^ (Fabaceae, BCN 32177)	garrofa (fruit)	Grain	Direct ingestion	Internal	Antidiarrhoeal	1	0	AE (0.006, 0.003)

*Citrus limon* (L.) Burm. f.^+^ (Rutaceae, BCN 46853)	llimoner, llimonera, llimona (fruit)	Fruit juice	Direct application	External	Ocular antiseptic	1	0	AE (0.006, 0.01)
			Direct ingestion	Internal	Internal antiseptic	1	1	MA (0.024, 0.01)
		Fruit (epicarp)	Medicinal soup (boiled rice with lemon peel)	Internal	Antidiarrhoeal	1	0	AT (0.017, 0.01)

*Clematis flammula* L.* (Ranunculaceae, BCN 29856)	santjoanet, vidauba	No data	Poultice	External	For swine erysipelas	1	0	MA (0.024, 0.003)

*Cucurbita maxima* Duch. in Lam. (Cucurbitaceae, BCN-S 1499)	rabequet (fruit)	Fruit	Ointment	External	Anti-inflammatory	1	1	AE (0.006, 0.003)

*Cucurbita pepo* L.^+^ (Cucurbitaceae, BCN 24757)	carbassa	Fruit	Ointment	External	Antiseptic	1	1	AT (0.017, 0.003)

*Daphne gnidium* L. (Thymelaeaceae^+^, BCN 29687)	tintorell, matapoll	Stem	Direct application	External	Vulnerary	1	0	AE (0.01, 0.01)
					Antidiarrhoeal	1	0	AE, FO (0.07, 0.01)

Diplotaxis* erucoides (L.) DC. (Brassicaceae, BCN 29861)	cap blanc	Flowered aerial part	Direct ingestion	Internal	Galactofugue	1	0	AE (0.006, 0.003)

Dracunculus* vulgaris Schott. (Araceae, BCN 24765)	herba escurçonera	Bulb	Embrocation	External	Antitoxic	2	2	AT (0.05, 0.01)
		Flower	Direct application	External	Antitoxic	1	1	AT

Eriobotrya* japonica (Thunb.) Lindl. (Rosaceae, BCN 66823)	nisprero, nispro (fruit)	Leaf	Decoction	Internal	Antidiarrhoeal	2	4	MA (0.05, 0.007)

*Eryngium campestre* L. (Apiaceae, BCN 31274)	espinacal	Flowered aerial part	Ointment	External	Antiophidian	1	0	AE (0.006, 0.03)
		Root	Liniment	External	Antiophidian	2	2	AT (0.12, 0.03)
		Aerial part	Direct application	External	Antiophidian	4	4	AT
					Antialgic	1	0	AT

*Eryngium bourgatii* L.* (Apiaceae, BCN 24881)	espinacal	Root	Liniment	External	Antiophidian	1	1	AT (0.08, 0.02)
		Aerial part	Direct application	External	Antiophidian	4	4	AT

*Eucalyptus globulus* Labill.^+^ (Myrtaceae, BCN 29696)	eucalipto, calipto	Leaf	Vapour	Internal	Anticatarrhal	2	47	AE (0.006, 0.007), FO (0.07, 0.007)

*Foeniculum vulgare* Mill. (Apiaceae, BCN 24888)	fonoll	Aerial part	Direct ingestion	Internal	Antidiarrhoeal	1	0	MA (0.02, 0.003)

*Fraxinus excelsior* L. (Oleaceae, BCN 24890)	freixe	Cortical parenchyma	Liniment (decoction with *Bryonia cretica*)	External	To heal wounds	1	1	AT (0.03, 0.007)
			Bath (decoction with *Quercus pubescens*)	External	To heal wounds	1	1	AT

*Gentiana acaulis* L.^+^ (Gentianaceae^+^, BCN 24892)	genciana	No data	No data	External	Antidermatose	1	0	AT (0.02, 0.003)

*Gentiana lutea* L.* (Gentianaceae^+^, BCN 24893)	genciana	Root	Bath	External	Vulnerary	1	0	AT (0.02, 0.003)

*Geranium rotundifolium* L. (Geraniaceae, BCN 29701)	cicuta	Aerial part	Ointment	External	Vulnerary	1	1	AE (0.006, 0.003)

*Globularia alypum* L. (Globulariaceae*, BCN 53082)	cossiada	Tender aerial part	Direct ingestion	Internal	Anorexigen	1	0	MA (0.02, 0.003)

*Herniaria hirsuta* L.* subsp. *cinerea* (DC.) Arcang. (Caryophyllaceae, BCN 23330)	trencapedra	Aerial part	Tisane	Internal	Diuretic	2	5	MA (0.05, 0.003)

*Herniaria glabra* L.^+^ (Caryophyllaceae, BCN 24901)	herba de mil granes, herba de les mil granes	Aerial part	Tisane	Internal	Antiicteric	1	0	AT (0.02, 0.003)

*Hypericum perforatum* L. (Clusiaceae, BCN 29874)	herba de Sant Joan	Flowered aerial part	Ointment	External	For mastitis	1	0	AE (0.006, 0.003)

*Juniperus communis* L. subsp. *alpina* (Suter) Čelak* (Cupressaceae, BCN 24910)	ginebre, ginebró, oli de ginebre	Fruit	Ointment	External	Antidermatose	3	2	AT (0.05, 0.01)

*Juniperus communis *L. subsp. *communis *(Cupressaceae, BCN 29878)	ginebre	Fruit	Ointment	External	Anti-inflammatory/ Antialgic/ Antiecchymotic	1	0	AE (0.01, 0.007)
					Vulnerary	1	0	AE

*Juniperus oxycedrus* L.^+^ (Cupressaceae, BCN 29879)	càdec, ginebró	Fruit	Ointment	External	Vulnerary	1	0	AE (0.03, 0.02)
					Demulcent	1	0	AE
					Antifungal	1	0	AE
					Internal antiseptic	1	0	AE
					For a sprained leg	1	0	AE

*Lavandula latifolia* Medic.^+^ (Lamiaceae, BCN 29882)	espígol	Flowered stem	Direct application	External	Antiophidian	1	0	AE (0.006, 0.003)

Lilium* pyrenaicum Gouan (Liliaceae, BCN 24918)	consolta, consolta groga	Bulb	Liniment	External	To heal wounds	1	1	AT (0.03, 0.007)
			Poultice	External	To heal wounds	1	1	AT

*Linum usitatissimum* L. (Linaceae, BCN 47281)	lli, llinet, oli de llinosa (product)	Grain	Ointment	External	Vulnerary	1	0	AE (0.01, 0.007)
			Enema	Internal	Laxative	1	6	AE

Lithospermum* officinale L. (Boraginaceae, BCN 24922)	herba pedrera, mill del sol, tabac	Aerial part with fruits	Tisane	Internal	Postpartum coadjuvant	1	0	AT (0.006, 0.003)

*Lythrum salicaria* L.^+^ (Lythraceae, BCN 29724)	tapaculs	Flowered aerial part	Tisane	Internal	Antidiarrhoeal	3	3	AE (0.02, 0.01)

*Malva sylvestris* L. (Malvaceae, BCN 24924)	malva	No data	Tisane	Internal	Anti-inflammatory	1	4	FO (0.07, 0.003)
		Flower	Decoction	Internal	Antiseptic	2	1	AT (0.13, 0.03)
		Flowered aerial part	Tisane (with *Parietaria officinalis*)	Internal	Postpartum coadjuvant	2	1	AT
			Tisane (with *Parietaria officinalis, Plantago lanceolata*, and *Plantago major*)	Internal	Postpartum coadjuvant	1	0	AT
					Diuretic	1	0	AT
			Tisane (with *Umbilicus rupestris*)	Internal	Purgative	1	0	AT
			Enema (with *Parietaria officinalis*)	Internal	Purgative	1	0	AT

*Marrubium vulgare* L. (Lamiaceae, BCN 29726)	herba del mal roig	Aerial part	Tisane	Internal	For swine erysipelas	1	0	AE (0.006, 0.003)

*Matricaria recutita* L.^+^ (Asteraceae, BCN 29890)	camamilla	Inflorescence	Direct ingestion	Internal	Digestive	1	28	AE (0.01, 0.007)
					Gastric and intestinal anti-inflammatory	1	13	AE

*Mentha spicata* L.* (Lamiaceae, BCN 29995)	menta del consol	Tender aerial part	Tisane	Internal	Postpartum coadjuvant	1	0	AE (0.006, 0.003)

*Mentha suaveolens* Ehrh.^+^ (Lamiaceae, BCN 29994)	herbasana	Aerial part	Tisane	Internal	Antihelminthic	1	1	MA (0.02, 0.003)

*Mercurialis annua* L. (Euphorbiaceae, BCN 29896)	murcarol	Aerial part	Tisane	Internal	Laxative	2	2	AE (0.02, 0.01)
			Direct ingestion	Internal	Laxative	1	0	AE

*Meum * ^+^ * athamanticum* Jacq. subsp. *athamanticum* (Apiaceae, BCN 24933)	herba del meu, meu	Root	Tisane (decoction with *Senecio leucophyllus*)	Internal	Postpartum coadjuvant (antiseptic)	13	1	AT (0.23, 0.05)
		Whole plant	Tisane (decoction with *Saxifraga longifolia*)	Internal	Postpartum coadjuvant	1	0	AT

*Nicotiana rustica *L.^+^ (Solanaceae, BCN 46839)	tabac pota	Leaf	Direct ingestion	Internal	Emetic	1	1	FO (0.14, 0.007)

*Ocimum basilicum* L.^+^ (Lamiaceae, BCN 29897)	fàbrega	Aerial part	Tisane (bath)	External	Puerperal antiseptic	1	0	AE (0.01, 0.007)
					External antiseptic	1	0	AE

*Olea europaea* L. var. *europaea * (Oleaceae, BCN 29898)	olivera, oli d'oliva (product)	Fruit	Ointment	External	For mastitis	1	0	AE (0.06, 0.04)
					Antiscabby	1	0	AE
					Vulnerary	1	0	AE
					Cicatrizing	4	0	AE, FO (0.07, 0.04)
					Antiseptic	1	0	AE
			Direct application	External	Haemostatic	1	0	AT (0.03, 0.04)
			Tablets	Internal	Salutiferous	1	1	AE
			Poultice	External	Insect repellent	3	0	AT, MA (0.05, 0.04)

*Origanum * ^+^ vulgare* L. (Lamiaceae, BCN 29742)	orenga	Flowered stem	Direct ingestion	Internal	Postpartum coadjuvant	1	0	AE (0.006, 0.007)
		Aerial part	Tisane	Internal	Internal antiseptic	1	2	FO (0.071, 0.007)

*Oryza sativa* L. (Poaceae, BCN 39208)	arròs	Grain	Tisane (boiled rice with lemon peel)	Internal	Antidiarrhoeal	1	2	AT (0.02, 0.003)

*Parietaria officinalis* L. subsp. *judaica* (L.) Béguinot (Urticaceae, BCN 29745)	blet de paret, cama-roja	Aerial part	Boiled plant and its decoction	Internal	Laxative	1	1	AE (0.006, 0.003)

*Parietaria officinalis* L. (Urticaceae, BCN 24942)	blet, blet de paret	Aerial part	Decoction (with *Malva sylvestris*)	Internal	Antiseptic	2	2	AT (0.3, 0.05)
			Medicinal broth (with *Beta vulgaris*, bread and olive oil)	Internal	Postpartum coadjuvant (emollient)	9	1	AT
			Tisane (decoction with *Malva sylvestris*)	Internal	Postpartum coadjuvant	1	0	AT
					Diuretic	1	0	AT
					Purgative	1	1	AT
			Enema	Internal	Purgative	1	1	AT

*Petroselinum crispum* (Mill.) Hill^+^ (Apiaceae, BCN 29905)	julivert	Leaf	Direct ingestion	Internal	Aphrodisiac	1	0	AE (0.01, 0.01)
		Whole plant	Direct application	Internal	Aphrodisiac	1	0	AT (0.02, 0.01)
		Root	Poultice	External	Antiparotid (for mumps)	1	0	AE

*Pimpinella anisum* L. (Apiaceae, BCN 47278)	anís (fruit)	Fruit	Elixir	Internal	Ruminant antistatic	1	0	AE (0.006, 0.003)

*Pinus halepensis* Mill.^+^ (Pinaceae, BCN 29826)	pi, pi bord	Bark	Direct application	External	Epithesis	1	0	AE (0.006, 0.007)
		Resin	Ointment	External	External antiseptic	1	3	MA (0.02, 0.007)

*Pinus* sp. (Pinaceae)	pega negra (product)	Resin	Ointment	External	Puerperal antiseptic	1	0	AE (0.01, 0.007)
			Patch	External	For a sprained leg	1	0	AE

*Pinus sylvestris* L.^+^ (Pinaceae, BCN 27259)	pi, pi roig, pega negra (product)	Resin	Poultice	External	Epithesis	1	0	AT (0.02, 0.003)

*Plantago lanceolata* L. (Plantaginaceae, BCN 24949)	plantatge, plantatge estret, plantatge llarg	Leaf	Tisane (with *Parietaria officinalis*)	Internal	Postpartum coadjuvant (diuretic)	1	0	AT (0.02, 0.003)

*Plantago major* L. (Plantaginaceae, BCN 24950)	plantatge, plantatge ample, plantatge rodó	Leaf	Tisane (with *Parietaria officinalis*)	Internal	Postpartum coadjuvant (diuretic)	1	0	AT (0.02, 0.003)

*Pteridium aquilinum* (L.) Kuhn (Polypodiaceae, BCN 46068)	falguera	Frond	Direct application	External	For flea infestation	1	1	AE (0.006, 0.003)

*Quercus ilex* L. subsp. *ilex * ^+^ (Fagaceae, BCN 29932)	alzina, aulina	Bark	Tisane (bath)	External	Vulnerary	6	2	AE (0.05, 0.04)
			Tisane	Internal	Antidiarrhoeal	3	0	AE
			Bath	External	Antiseptic	3	1	AT (0.05, 0.04)

*Quercus petraea* (Matt.) Liebl.* (Fagaceae, BCN 24964)	roure	Internal bark	Bath (with *Fraxinus excelsior*)	External	Antiseptic	3	0	AT (0.07, 0.01)
			Bath	External	Cicatrizing	1	1	AT

Ramonda* myconi (L.) Reichenb. (Gesneriaceae, BCN 24965)	orella d'ós	Aerial part	Tisane	Internal	Postpartum coadjuvant (antiseptic)	1	0	AT (0.02, 0.003)

*Ranunculus parnassifolius* L.* (Ranunculaceae, BCN 24967)	herba del mal gra	Whole plant	Liniment	External	Antigangrenous	1	1	AT (0.2, 0.05)
			Liniment, bath	External	Antiseptic	4	5	AT
			Liniment, bath	External	Resolutive	4	5	AT
			Liniment, poultice	External	Antineoplastic	5	6	AT

*Rosmarinus officinalis* L. (Lamiaceae, BCN 29937)	romaní	Flowered stem	Tisane	Internal	Postpartum coadjuvant	1	0	AE (0.006, 0.007)
		Aerial part	Direct ingestion	Internal	Antidiarrhoeal	1	0	FO (0.07, 0.007)

*Ruta chalepensis* L. (Rutaceae, BCN 29940)	ruda	Aerial part	Direct ingestion	Internal	Carminative	1	0	AE (0.006, 0.04)
					Ruminant antistatic	2	0	MA (0.07, 0.04)
			Direct application	External	Postpartum coadjuvant (antiseptic)	3	0	AT (0.10, 0.04)
					Tranquilizer	1	0	FO (0.14, 0.04)
					Flea repellent	1	0	FO
			Embrocation (with *Bryonia cretica* or *Umbilicus rupestris*)	External	To heal wounds	1	2	AT
			Liniment	External	To heal wounds	1	2	AT
					Anti-inflammatory	1	0	AT
			No data	Internal	Antihelminthic	1	10	MA

*Sambucus nigra* L. (Caprifoliaceae, BCN 29943)	sabuquer, sabuc, flor de sabuc	Inflorescence	Fumigation	External	For mastitis	1	0	AE (0.006, 0.03)
		Flower	Tisane (with *Achillea ptarmica*)	Internal	Antidiarrhoeal	2	1	AT (0.15, 0.03)
			Tisane	Internal	Intestinal antiseptic	2	14	AT
			Essence (with *Achillea ptarmica*)	Internal	Antidiarrhoeal	2	1	AT
			Essence	Internal	Intestinal antiseptic	2	14	AT
		Fruit	Syrup	Internal	Intestinal antiseptic	1	9	AT

*Santolina chamaecyparissus* L.* (Asteraceae, BCN 29944)	camamilla	Flower head	Tisane	Internal	Internal antiseptic	1	5	FO (0.07, 0.01)
			Bath	External	Ocular antiseptic	2	8	MA (0.07, 0.01)
			Enema	Internal	Puerperal antiseptic	1	0	MA

*Saxifraga longifolia* Lap.* (Saxifragaceae)	corona de rei	Aerial part	Tisane (with *Ramonda myconi* or *Meum athamanticum*)	Internal	Postpartum coadjuvant (antiseptic)	1	0	AT (0.02, 0.003)

*Saxifraga paniculata* Mill.^+^ (Saxifragaceae, BCN 24992)	corona de rei	Aerial part	Tisane	Internal	Intestinal anti-inflammatory	1	1	AT (0.03, 0.007)
					Postpartum coadjuvant	1	0	AT

*Scabiosa columbaria* L.* (Dipsacaceae, BCN 24993)	escabiosa, escapiosa, herba d'escapiosa	Aerial part	Tisane	Internal	Antiparasitic	1	0	AT (0.02, 0.003)

*Sedum * ^+^ * sediforme* (Jacq.) Pau* (Crassulaceae, BCN 29792)	mort-i-viu, herba de Sant Pere	Leaf	Poultice	External	Anti-inflammatory/ Antialgic/ Antiecchymotic	1	0	AE (0.01, 0.007)
					Antiseptic	1	0	AE

*Sedum * ^+^ * telephium* L.* (Crassulaceae, BCN 24995)	arròs de paret, bàlsam, herba grassa, matafocs	Leaf	Ointment	External	Antidermatose	2	2	AT (0.03, 0.007)

*Sempervivum montanum* L.* (Crassulaceae, BCN 24996)	matafoc de muntanya	Aerial part	Liniment	External	Antidermatose	1	0	AT (0.02, 0.003)

*Senecio leucophyllus* L.* (Asteraceae, BCN 24998)	herba blanca	Aerial part	Tisane (decoction with *Meum athamanticum*)	Internal	Postpartum coadjuvant	1	0	AT (0.02, 0.003)

*Solidago virgaurea* L.* (Asteraceae, BCN 25007)	vara d'or	Flowered aerial part	Tisane	Internal	Antitoxic	1	0	AT (0.02, 0.003)

*Sonchus oleraceus *L. (Asteraceae, BCN 29953)	llipsó, lletissó	Aerial part	Direct ingestion	Internal	Galactogenous	1	0	AE (0.006, 0.01)
			Liniment (with *Senecio vulgaris*)	External	Anti-inflammatory	1	1	AT (0.05, 0.01)
					Antigotose	1	1	AT
			Liniment	External	Vulnerary	1	1	AT

*Sorbus aucuparia* L.* (Rosaceae, BCN 25009)	moixera de guilla	Aerial part	Direct application	External	Antiviral	1	0	AT (0.02, 0.003)

*Tanacetum parthenium* (L.) Schultz Bip.^+^ (Asteraceae, BCN 29960)	tanarida, herba del remuc, camamilla, camamilla amargant, camamilla borda, camamilla de parets, danarida, herba danarida	Flowered aerial part	Tisane	Internal	Ruminant antistatic	3	0	AE (0.08, 0.08)
			Direct ingestion	Internal	Ruminant antistatic	7	0	AE
			Tisane	Internal	Antiicteric	1	0	AE
				Internal	Antidiarrhoeal	2	0	AT (0.15, 0.08)
				Internal	Digestive	2	3	AT
				Internal	Antinausea	1	1	AT
				Internal	Unknown	5	0	AT
			Tablets	Internal	Ruminant antistatic	2	0	AE
				Internal	Antiicteric	1	0	AE

Taraxacum* officinale Weber in Wiggers (Asteraceae, BCN 25948)	dent de lleó	Leaf	Direct ingestion	Internal	Bone reinforcer	1	1	AE (0.006, 0.003)

*Thymus vulgaris* L. (Lamiaceae, BCN 29961)	farigola, frígula, frigola	Flowered stem	Tisane (bath)	External	Antiseptic	5	40	AE (0.06, 0.03)
					Ocular antiseptic	2	10	AE
					Vulnerary	1	8	AE
			Direct ingestion	Internal	Carminative	1	1	AE
		Tender aerial part	Tisane	Internal	Postpartum coadjuvant	1	0	AE

*Thymus serpyllum *L. subsp. *chamaedrys* (Fries) Čelak^+^ (Lamiaceae, BCN 25019)	farigoleta, farigoleta de muntanya, serpó	Flowered aerial part	Tisane	Internal	Antidiarrhoeal	1	1	AT (0.02, 0.003)

*Thymus serpyllum* L. subsp. *nervosus* (Gay ex Willk.) Nyman* (Lamiaceae, BCN 25020)	farigoleta, farigola de muntanya, farigola de pastor, farigolet, xerpoll	Flowered aerial part	Tisane	Internal	Antidiarrhoeal	3	1	AT (0.05, 0.01)

*Triticum aestivum* L. (Poaceae, BCN 29963)	blat	Bran	Poultice	External	Diuretic	1	0	AE (0.006, 0.003)

*Umbilicus rupestris* (Salisb.) Dandy^+^ (Crassulaceae, BCN 25029)	barret de paret	Aerial part	Liniment (boiled with olive oil and *Allium sativum*, *Althaea officinalis*, *Bryonia cretica*, *Lilium pyrenaicum*,* Ruta chalepensis*, *Petroselinum crispum*,* Sedum* sp.)	External	To heal wounds	1	1	AT (0.05, 0.01)
			Direct application	External	To heal wounds	1	1	AT
			Tisane	Internal	Purgative	1	0	AT

*Urginea maritima* (L.) Baker (Liliaceae, BCN 58049)	ceba marina	Bulb	Maceration in oil	External	Insect repellent	2	0	MA (0.05, 0.007)

*Urtica dioica* L. (Urticaceae, BCN 29814)	ortigues	Aerial part	Direct application	External	Aphrodisiac	1	0	AE (0.006, 0.003)

*Veratrum album* L. (Liliaceae, BCN 25034)	veladre	Aerial part	Tisane	External	Antiparasitic (for mange in sheep)	1	0	AT (0.05, 0.01)

*Verbascum sinuatum* L. (Scrophulariaceae, BCN 29967)	trepó	Flower	Tisane	Internal	Antidiarrhoeal	1	1	AE (0.006, 0.003)

*Vicia faba* L. (Fabaceae, BCN 46826)	favera	Grain	Direct ingestion	Internal	For studs' fertility enhancement	1	0	AE (0.006, 0.003)

*Vitis vinifera* L. (Vitaceae, BCN 29972)	vinya, vi (product), vinagre (product)	Fruit juice	Vinegar	External	Anticatarrhal	1	1	AE (0.02, 0.02)
					Diuretic	1	0	AE
					Antiseptic	1	1	AE
					Tranquilizer	1	0	FO (0.14, 0.02)
			Medicinal wine	Internal	Restorative	1	0	MA (0.02, 0.02)
					Blue comb	1	0	FO

**Table 2:** General data on the territories studied, data concerning ethnoveterinary and related aspects, and ethnobotanical indexes.

	AE	AT	FO	MA	Total
General Data					
Extension (km^2^)	1358	294	81.2	238	1971.2
Population	118718	4526	9147	31764	164155
Number of total informants	179	60	14	42	295
Number of informants with veterinary reports	46	41	4	13	104
Number of taxa in the flora of the territory	1650^(1)^	1600^(2)^	574^(3)^	780^(4)^	—
Total of livestock (includes cattle, sheep, goats, pigs, horses, and poultry)	2345153^(5)^	28088^(6)^	3229911^(7)^		5525414
Ethnoveterinary and other ethnobotanical data					
Number of taxa with veterinary uses	49	49	11	17	100
Number of species with veterinary uses	48	47	11	17	96
Number of veterinary use reports	106	146	16	28	306
Number of coincidental human and animal medicine use reports	33	104	4	16	157
Number of local Catalan names of plants with veterinary uses	55	70	11	18	154
Number of botanical families containing plants with veterinary uses	31	27	10	13	41
Number of veterinary reports/number of informants with veterinary reports	2.30	3.56	4.00	2.15	2.94
Number of animal feed taxa	73	21	14	16	—
Number of human medicinal taxa	334	220	92	117	—
Percentage of veterinary uses coincidental with human medicinal ones	30.1	52.2	38.5	50	42.9
Ethnobotanical indexes					
Ethnoveterinaricity index (EvI)	0.03	0.03	0.02	0.02	—
Informant consensus factor (F_IC_)	0.54	0.68	0.33	0.40	0.67
Average index of cultural importance (CI) calculated per area	0.01	0.05	0.10	0.04	0.04
Average index of cultural importance (CI) calculated for all studied areas	0.01	0.01	0.02	0.01	0.01

^(1)^[77]; ^(2)^J. Vigo (pers. comm.); ^(3)^[79]; ^(4)^[80]; ^(5)^[81]; ^(6)^[81]; ^(7)^Data for the whole Balearic archipelago [73].

## References

[1] Wanzala W, Zessin KH, Kyule NM, Baumann MPO, Mathias E, Hassanali A (2005). Ethnoveterinary medicine: a critical review of its evolution, perception, understanding and the way forward. *Livestock Research for Rural Development*.

[2] Mathias E, McCorkle CM, Schillhorn Van Veen TW, McCorkle CM, Mathias E, Schillhorn Van Veen TW (1996). Introduction: ethnoveterinary research and development. *Ethnoveterinary Research and Development*.

[3] Mathias ER, Martin M, McCorkle CM (2001). *Ethnoveterinary Medicine: An Annotated Bibliography of Community Animal Healthcare*.

[4] Van den Eynden V, Vernemmen P, Van Damme P (1992). *The Ethnobotany of the Topnaar*.

[5] Moerman DE (1998). *Native American Ethnobotany*.

[6] Brüschweiler S

[7] Rigat M, Garnatje T, Vallès J (2006). *Plantes i gent. Estudi etnobotànic de l’Alta Vall del Ter*.

[8] Ghosh Ch, Das AP, Das AP, Pandey AK (2007). Plants of ethnobotanical significance for the tea garden workers in Teral and Duars of Darjeeling in West Bengal, India. *Advances in Ethnobotany*.

[9] Pieroni A, Pardo-de-Santayana M, Pieroni A, Puri RK (2010). People and plants in Lëpushë: traditional medicine, local foods and post-communism in a northern Albanian village. *Ethnobotany in the new Europe. People, health and wild plant resources*.

[10] Geerlings E (2001). *Sheep husbandry and Ethnoveterinary knowledge of Raika sheep pastoralists in Rajasthan, India*.

[11] Lans C, Harper T, Georges K, Bridgewater E (2001). Medicinal and ethnoveterinary remedies of hunters in Trinidad. *BMC Complementary and Alternative Medicine*.

[12] Lans C, Turner N, Khan T, Brauer G, Boepple W (2007). Ethnoveterinary medicines used for ruminants in British Columbia, Canada. *Journal of Ethnobiology and Ethnomedicine*.

[13] Abbas B, Al-Qarawi AA, Al-Hawas A (2002). The ethnoveterinary knowledge and practice of traditional healers in Qassim region, Saudi Arabia. *Journal of Arid Environments*.

[14] Tabuti JRS, Dhillion SS, Lye KA (2003). Ethnoveterinary medicines for cattle (*Bos indicus*) in Bulamogi county, Uganda: plant species and mode of use. *Journal of Ethnopharmacology*.

[15] Pearson W Ethnoveterinary medicine: the science of botanicals in equine health and disease.

[16] Muhammad G, Khan MZ, Hussain MH, Iqbal Z, Iqbal M, Athar M (2005). Ethnoveterinary practices of owners of pneumatic-cart pulling camels in Faisalabad City (Pakistan). *Journal of Ethnopharmacology*.

[17] Mitra S, Mukherjee SK, Das AP, Pandey AK (2007). Plants used as ethnoveterinary medicine in Uttar and Dakshin Dinajpur Districts of West Bengal, India. *Advances in Ethnobotany*.

[18] Pieroni A, Giusti ME, de Pasquale C (2006). Circum-Mediterranean cultural heritage and medicinal plant uses in traditional animal healthcare: a field survey in eight selected areas within the RUBIA project. *Journal of Ethnobiology and Ethnomedicine*.

[19] Gradé JT (2008). *Ethnoveterinary knowledge in pastoral Karamoja, northern Uganda*.

[20] Gradé J, Weladji R, Tabuti J, Van Damme P (2009). Healer-driven ethnoveterinary knowledge diffusion among semi-nomadic pastoralists in Karamoja, Uganda. *Afrika Focus*.

[21] Katerere DR, Luseba D (2010). *Ethnoveterinary Botanical Medicine. Herbal Medicines for Animal Health*.

[22] Phondani PC, Maikhuri RK, Kala CP (2010). Ethnoveterinary uses of medicinal plants among traditional herbal healers in Alaknanda catchment of Uttarakhand, India. *African Journal of Traditional, Complementary and Alternative Medicines*.

[23] Martinez GJ, Lujan MC (2011). Medicinal plants used for traditional veterinary in the Sierras de Cordoba (Argentina): an ethnobotanical comparison with human medicinal uses. *Journal of Ethnobiology and Ethnomedicine*.

[24] Mishra D (2011). Ethnoveterinary Practices and use of herbal medicines for treatment of skin diseases in cattle: a study in Polsara Block, Ganjam District, Orissa, India. *Veterinary World*.

[25] Offiah NV, Makama S, Elisha IL (2011). Ethnobotanical survey of medicinal plants used in the treatment of animal diarrhoea in Plateau State, Nigeria. *BMC Veterinary Research*.

[26] Brisebarre AM, McCorkle CM, Mathias E, Schillhorn Van Veen TW (1996). Tradition and modernity: French shepherds’ use of medicinal bouquets. *Ethnoveterinary Research and Development*.

[27] Iqbal Z, Jabbar A, Katerere DR, Luseba D (2010). Inventory of traditional veterinary botanicals from around the world. *Ethnoveterinary Botanical Medicine. Herbal Medicines for Animal Health*.

[28] Pardo-de-Santayana M, Pieroni A, Puri RK (2010). *Ethnobotany in the New Europe. People, Health and Wild Plant Resources*.

[29] Pieroni A (1999). *Herbs, Humans and Animals / Erbe, uomini e bestie*.

[30] Viegi L, Pieroni A, Guarrera PM, Vangelisti R (2003). A review of plants used in folk veterinary medicine in Italy as basis for a databank. *Journal of Ethnopharmacology*.

[31] Bonet MA, Vallès J (2007). Ethnobotany of Montseny biosphere reserve (Catalonia, Iberian Peninsula): plants used in veterinary medicine. *Journal of Ethnopharmacology*.

[32] Belda A, Martínez-Pérez JE, Martín C, Peiró V, Seva E (2010). Plants used to capture and sustain wild finches (Fringillidae) in southeast Spain. *Economic Botany*.

[33] Akerreta S, Calvo MI, Cavero RY (2010). Ethnoveterinary knowledge in Navarra (Iberian Peninsula). *Journal of Ethnopharmacology*.

[34] Benítez G, González-Tejero MR, Molero-Mesa J (2012). Knowledge of ethnoveterinary medicine in the Province of Granada, Andalusia, Spain. *Journal of Ethnopharmacology*.

[35] González JA, García-Barriuso M, Amich F (2011). Ethnoveterinary medicine in the Arribes del Duero, western Spain. *Veterinary Research Communications*.

[36] Agelet A, Vallès J, Pieroni A (1999). Vascular plants used in ethnoveterinary in Pallars (Pyrenees, Catalonia, Iberian Peninsula). *Herbs, Humans and Animals / Erbe, uomini e bestie*.

[37] Rigat M, Bonet MA, Garcia S, Garnatje T, Vallés J (2009). Ethnobotany of food plants in the high river Ter valley (Pyrenees, Catalonia, Iberian Peninsula): non-crop food vascular plants and crop food plants with medicinal properties. *Ecology of Food and Nutrition*.

[38] Parada M, Carrió E, Vallès J (2011). Ethnobotany of food plants in the Alt Empordà region (Catalonia, Iberian peninsula). *Journal of Applied Botany and Food Quality*.

[39] Rigat M, Garnatje T, Vallès J (2011). Plant biodiversity in Pyrenean homegardens (Catalonia, Iberian peninsula): current state of a mountain agroecosystem. *Acta Botanica Gallica*.

[40] Torres M (1999). *Antropologia d’Eivissa i Formentera. Herbes, pastors, ses matances*.

[41] Carrió E, Mayans M, Vallès J (2011). Abans que es pins facin magranes i ses figueres melons. Primeres dades de dues recerques etnobotàniques a Formentera i Mallorca. *Mètode*.

[42] (Meteorological Service of Catalonia) SMC Generalitat de Catalunya. http://www.meteo.cat/.

[43] Folch R (1986). *La vegetació dels Països Catalans*.

[44] Vigo J (2010). *L’alta muntanya catalana. Flora i vegetació*.

[45] (Balearic Islands Statistical Institute) IBESTAT Govern de les Illes Balears. http://www.ibestat.cat/.

[46] (Meteorological National Agency) AEMET Ministerio de Agricultura, Alimentación y Medio Ambiente, Spain. http://www.aemet.es/.

[47] Llorens L, Gil L, Tébar FJ (2007). *La vegetació de l’illa de Mallorca: bases per a la interpretació i gestió d’hàbitats*.

[48] (Statistical National Institute) INE http://www.ine.es/.

[49] Pujadas JJ, Comas D, Roca J (2004). *Etnografia*.

[50] de Albuquerque UP, Farias Paiva de Lucena R, Fernandes Cruz da Cunha LV (2008). *Métodos e Técnicas na Pesquisa Etnobotánica*.

[51] Nolan JM, Turner NJ, Anderson EN, Pearsall DM, Hunn ES, Turner NL (2011). Ethnobotany: the study of people - plants relationships. *Ethnobiology*.

[52] de Bolòs O, Vigo J (2001). *Flora dels Països Catalans*.

[53] de Bolòs O, Vigo J, Masalles RM, Ninot JM (2005). *Flora manual dels Països Catalans*.

[54] Fournier P (1953). *Flore illustrée des jardins et des parcs. Arbres, arbustes et fleurs de pleine terre*.

[55] Sánchez-Monge E (1991). *Flora Agricola. Taxonomía de las Magnoliofitas (Angiospermas) de interés agrícola, con excepción de las de aprovechamiento exclusivamente ornamental o forestal*.

[56] Carrió E, Parada M, Parada J Towards a database on ethnobotany of the Catalan linguistic area.

[57] Portères R (1970). *Cours d‘Ethno-botanique et Ethno-zoologie (1969-1970). Volume I, Ethno-botanique générale*.

[58] Trotter RT, Logan MH, Etkin NL (1986). Informant consensus: a new approach for identifying potentially effective medicinal plants. *Plants in Indigenous Medicine and Diet, Behavioural Approaches*.

[59] Tardío J, Pardo-de-Santayana M (2008). Cultural importance indices: a comparative analysis based on the useful wild plants of southern Cantabria (northern Spain). *Economic Botany*.

[60] Höft M, Barik SK, Lykke AM (1999). Quantitative ethnobotany. Applications of multivariate and statistical analyses in ethnobotany. *People and Plants Working Paper 6*.

[61] The R Project for Statistical Computing. http://www.r-project.com.

[62] Ruskey F, Weston M A survey of Venn Diagrams. http://www.combinatorics.org/Surveys/ds5/VennEJC.html.

[63] Villar L, Palacín JM, Calvo C, Gómez D, Montserrat G

[64] Houghton PJ, Osibogun IM (1993). Flowering plants used against snakebite. *Journal of Ethnopharmacology*.

[65] Ertug F, Pieroni A (1999). Plant, animal and human relationships in the folk medicine of Turkey. *Herbs, Humans and Animals / Erbe, uomini e bestie*.

[66] Parada M, Carrió E, Bonet MA, Vallès J (2009). Ethnobotany of the Alt Empordà region (Catalonia, Iberian Peninsula). Plants used in human traditional medicine. *Journal of Ethnopharmacology*.

[67] Johns T, Kokwaro JO, Kimanani EK (1990). Herbal remedies of the Luo of Siaya District, Kenya: establishing quantitative criteria for consensus. *Economic Botany*.

[68] González-Tejero MR, Casares-Porcel M, Sánchez-Rojas CP (2008). Medicinal plants in the Mediterranean area: synthesis of the results of the project Rubia. *Journal of Ethnopharmacology*.

[69] Reyes-García V, Vila S, Aceituno-Mata L (2010). Gendered homegardens: a study in three mountain areas of the Iberian Peninsula. *Economic Botany*.

[70] Font Quer P (1961). *Plantas medicinales. El Dioscórides renovado*.

[71] Rigat M, Bonet MA, Garcia S, Garnatje T, Vallès J (2007). Studies on pharmaceutical ethnobotany in the high river Ter valley (Pyrenees, Catalonia, Iberian Peninsula). *Journal of Ethnopharmacology*.

[72] Carrió E (2008). *Contribució al coneixement etnobotànic de Mallorca*.

[73] Desco Y, Mas L Estadístiques bàsiques de l’agricultura, la ramaderia i la pesca a les Illes Balears.

[74] Carrió E, Vallès J (2012). Ethnobotany of medicinal plants used in Eastern Mallorca (Balearic Islands, Mediterranean Sea). *Journal of Ethnopharmacology*.

[75] Akerreta S, Cavero RY, Calvo MI (2007). First comprehensive contribution to medical ethnobotany of Western Pyrenees. *Journal of Ethnobiology and Ethnomedicine*.

[76] Heinrich M, Ankli A, Frei B, Weimann C, Sticher O (1998). Medicinal plants in Mexico: Healers’ consensus and cultural importance. *Social Science and Medicine*.

[77] Leonti M, Vibrans H, Sticher O, Heinrich M (2001). Ethnopharmacology of the Popoluca, Mexico: an evaluation. *Journal of Pharmacy and Pharmacology*.

[78] Ragupathy S, Steven NG, Maruthakkutti M, Velusamy B, Ul-Huda MM (2008). Consensus of the ’Malasars’ traditional aboriginal knowledge of medicinal plants in the Velliangiri holy hills, India. *Journal of Ethnobiology and Ethnomedicine*.

[79] Gil Vives L, Llorens García L (2004). Biogeographical analysis of the flora of Formentera (Balearic Island, Spain). *Lazaroa*.

[80] Garcías L Contribució a la flora balear (I-IV, IX). Plantes dels voltants d’Artà i Capdepera. *Butlletí de la Institució Catalana d’Història Natural, 1905 – 1949*.

[81] (Catalonia Statistical Institute) IDESCAT Generalitat de Catalunya. http://www.idescat.cat/.

[82] Asseldonk TV, Katerere DR, Luseba D (2010). Ethnoveterinary medical practice in the European Union (EU). A case study of the Netherlands. *Ethnoveterinary Botanical Medicine. Herbal Medicines for Animal Health*.

